# Current Knowledge on the Importance of Selenium in Food for Living Organisms: A Review

**DOI:** 10.3390/molecules21050609

**Published:** 2016-05-10

**Authors:** Marek Kieliszek, Stanisław Błażejak

**Affiliations:** Faculty of Food Sciences, Department of Biotechnology, Microbiology and Food Evaluation, Warsaw University of Life Sciences—SGGW, Nowoursynowska 159 C, 02-776 Warsaw, Poland; stanislaw_blazejak@sggw.pl

**Keywords:** selenium, cancer, food, nutrition

## Abstract

Selenium is one of the elements classified within the group of micronutrients which are necessary in trace amounts for the proper functioning of organisms. Selenium participates in the protection of cells against excess H_2_O_2_, in heavy metal detoxification, and regulation of the immune and reproductive systems as well. It also ensures the proper functioning of the thyroid gland. Selenium induces the occurrence of the selenoprotein synthesis process involved in the antioxidant defense mechanism of the organism. Recent years have brought much success in the studies on selenium. Anticarcinogenic properties of selenium against some cancers have been reported. Supplementation is increasingly becoming a solution to this problem. A large number of different supplementation methods are promoting studies in this area. Slight differences in the selenium content can result in excess or deficiency, therefore supplementation has to be done carefully and cautiously.

## 1. Introduction

The discovery of selenium by the Swedish chemist J.J. Berzelius in 1817 initiated studies evaluating the influence of the inorganic forms of this element on living organisms. Unexpectedly, in 1957, Schwartz and Folz demonstrated the protective effect of selenium on organisms. Thanks to these studies, selenium was included in a group of trace elements whose deficiencies in the diet may cause numerous diseases. Due to the biological activity of selenium and its importance in human and animal nutrition, this element has an impact on health improvement and the immune system [1,2].

Selenium deficiency in the diet may have an adverse effect on health. Dietary selenium deficiency affects 0.5–1 billion people in the world, and currently, in many countries there is an inadequate intake of this element [3]. According to the World Health Organization (WHO), the maximum daily intake of selenium should not exceed 70 μg/day [1]. One should strive to adhere to the recommended supply dosage as well as the upper tolerable intake limit of this element. Selenium doses above 400 μg/day to 700 μg/day may exert toxic actions. The average content of selenium in the daily diet is far from the recommended content of this element. The estimated content based on the typical household consumption ranges between 30 and 50 μg/day in various European countries [1,4,5,6,7,8].

In view of the very diverse range of selenium intakes, extensive educational programs providing information on the positive impact of this element on health should be carried out. However, its toxic properties should not be forgotten—especially considering its narrow therapeutic index, as its toxic dose starts at 400 μg/day. It should be noted that uncontrolled intake of products enriched with selenium may result in poisoning [9]. In Venezuela, studies have shown that the consumption of the fruit of the species *Lecythis ollaria* which accumulate huge amounts of selenium caused hair loss, diarrhea, and emesis in humans [10].

## 2. Selenium Contents of Foods

The content of selenium in foods is characterized by a great diversity. It depends on the concentration of selenium in the soil in a given geographical area, as well as the ability of plants to accumulate this element [11]. Moreover, other factors such as climatic conditions, cultivation and breeding methods, and methods of preparing food products also exert an effect. The content of selenium in a sampling of food products is presented in Table 1.

Selenium in food products most often occurs in combination with proteins, thus products with high protein content are typically characterized by a higher selenium content. These products include meat, fish, offal, and cereals [3]. In the meat products group, the selenium content ranges between 0.08 and 0.73 µg/g [6]. Fish of both marine (0.11−0.97 µg/g) and freshwater (0.18−0.68 µg/g) origin are rich in selenium [18]. The level of selenium in cereal products ranges from 0.01 to 0.55 µg/g [7]. In terms of dairy products, selenium levels are negatively correlated with fat content and range between 0.01 and 0.55 µg/g.

Fruits and vegetables contain small amount of selenium, ranging from 0.001 to 0.022 µg/g. This is caused by their low content of protein and high content of water. Extremely high levels of this element are nevertheless found in Brazil nuts and mushrooms [10]. Mushrooms contain substantial amounts of protein, in the range from 16.5% to 39% of dry matter, therefore their protein fractions exhibit high levels of organic selenium [19]. Common mushrooms (*Agaricus bisporus*) are among the most often studied mushrooms in selenium speciation studies. They are commonly eaten as a delicacy in Europe and the USA. In most plants, it is not possible to accumulate large amounts of selenium (*i.e*. selenium content rarely exceeds 100 µg/g). High concentrations of selenium have also been found in plants of the *Brassica* genus (broccoli, cabbage, cauliflower, and kohlrabi) [11,20].

Onion and garlic are a good source of selenium; they decrease the risk of cancer development. Moreover, the consumption of these plants does not cause excessive accumulation of selenium in tissues, or any other disorders [6]. The selenium in their composition occurs most commonly in the form of γ-glutamyl-Se-methylselenocysteine or Se-methylselenocysteine [21] (Table 2). Preparations enriched with macronutrients and trace elements are of particular importance. These are vitamin and mineral preparations, as well as those containing other essential nutrients. In these preparations, selenium occurs in the form of inorganic combinations (usually Se(IV)), or organic compounds (selenoamino acids). It is worth noting that preparations produced from yeasts are a rich source of selenium. In comparison with preparations containing inorganic selenium, selenium yeasts constitute valuable source of easily assimilable selenium [22].

## 3. Selenium Deficiency in the Diet

Selenium is an element whose trace amounts are essential for life, as proven in 1979 based on its cumulative function with vitamin E [23]. Increasing deficiency of selenium in different parts of the world leads to the occurrence of many pathological disorders. Particularly vulnerable to the adverse effects of selenium deficiency are patients suffering from phenylketonuria [24] or individuals with diet-related diseases. In addition, individuals exposed to specialized chemotherapy and individuals who have already undergone radiotherapy are vulnerable to decreased levels of this microelement in the organism [25].

Deficiency of selenium was confirmed in humans and animals inhabiting geographical regions where soils are characterized by low contents of this element [26]. The most serious consequences of selenium deficiency have been reported in a large part of China as well as Central and Eastern Siberia. It mainly results from insufficient supply of this micronutrient in the diet, individual culinary tastes of different social groups, or changes in the eating habits.

Selenium deficiency leads primarily to degeneration of many organs and tissues, resulting from decreased expression of selenoproteins, and thereby changes in the biological processes in which it participates [12]. Symptoms of selenium deficiency found in humans and animals are primarily disorders related to heart muscle and joints. Moderate deficiencies of this micronutrient may also have a negative impacts on human health, for example increasing the risk of infertility in men, prostate cancer, nephropathy, or the risk of the occurrence of neurological diseases [27]. In addition, selenium deficiency causes a dilated cardiomyopathy (Keshan disease) and endemic osteoarthropathy (Kashin-Beck disease) [12].

Kashin-Beck disease manifests itself by rheumatoid arthritis, shortened fingers and toes, or growth disorders of the organism. Oxidative damage of cartilage leads to deformation of the bone structure, known as degeneration (necrosis) of hyaline cartilage [28]. This disease mainly affects children aged between 5 and 13 years [6,29]. The combination of selenium and iodine deficiency constitutes a factor favoring the development of Kashin-Beck disease [30]. Another disease associated with a deficiency of selenium is Keshan. It is a juvenile cardiomyopathy and occurs mainly in young women of reproductive age and children aged between 2 and 10 years [31].

Deficiency of selenium may lead to the occurrence of other diseases, such as asthma—associated with impaired activity of glutathione peroxidase, promoted development of AIDS—deficiency causes its significant progression, impaired circulation, cardiac arrhythmia, stroke, or sudden infant death syndrome [32].

Selenium supplementation is an important issue. Among the methods of supplementation, one should distinguish the use of yeasts enriched with selenium as fodder components, enrichment of plants, as well as the addition of selenates directly to the fodder or oral administration of sodium selenite [13,33,34].

## 4. Consequences of Excess Content of Selenium Intake with the Diet

Excess selenium intake can lead to adverse effects, as described in 1930 when the occurrence of various diseases was observed after consumption of wild plants of the genus *Astragalus* by livestock [35]. Numerous cases of selenium excess in residents and animals living in a given geographical region are usually conditioned by large amounts of selenium in the soils. Animal grazing in areas where the dose of selenium is above 5 µg/g should be considered as dangerous to animals’ health. Selenium compounds are characterized by different degrees of toxicity. Inorganic sources of selenium exhibit higher toxicity as compared to the organic forms [17].

Excess of selenium in the diet causes chronic food poisoning symptoms such as vomiting, nausea, and diarrhea [36]. Acute exposure to high amounts of selenium leads to a general weakness of the organism, as well as neurological disorders [6]. In any case, toxicity of selenium is determined by many factors including the form of the occurrence of this element, ingested dose, physiological condition of the organism, as well as interaction of selenium with other diet components [37].

Chronic toxicity caused by an excess of selenium in living organisms leads to selenosis symptoms, which is manifested by hair loss, infertility, changes and fragility of fingernails or hooves, gastrointestinal upsets, skin rash, unpleasant “garlic” odor in exhaled air (dimethylselenide), and the occurrence of nervous system disorders [38,39]. Among other effects related to toxic doses of selenium, the presence of endocrine disruption in the synthesis of thyroid hormones, growth hormone (GH), and insulin-like growth factor (IGF-I) [6] can be included. Excessive concentration of selenium in serum and liver established at above 2 µg/g, is a symptom of severe toxicity. Particularly noteworthy is the occurrence of hematological abnormalities in blood [35].

Inhalation with selenium compounds, and especially highly toxic hydrogen selenide causes commonly observed symptoms of respiratory diseases, among others, chemical pneumonia and bronchitis [26]. Other symptoms include inflammation of pulmonary alveoli with pulmonary edema and hemorrhage [34], nausea, eye irritation, and headaches [12].

Consumption of *Lecythis ollaria* nuts containing large amounts of selenium (>5 mg/kg) led to an acute food poisoning episode in Venezuela [36]. In the case of livestock, fodder consumption in which selenium content is estimated between 5 and 50 µg/g results in the occurrence of hoove dystrophy in cattle and horses. The toxic effects of selenium on the organism are related to the production of free radicals causing DNA damage. Toxic effects of selenium are also associated with affinity towards thiol groups affecting disorder of the integrity of protein functions responsible for DNA repair [40]. High levels of selenium in the organism cause serious liver damage, decreased triiodothyronine [T3] concentration, and the loss of natural killer cells [6].

In China, scientific papers have reported that selenosis symptoms occurred with increased frequency in individuals who consumed excessive selenium at a dose exceeding 850 µg/day [26]. Analysis of the results carried out by Zwolak and Zaporowska [41] showed that consumption of selenium at doses up to 724 µg/day did not cause selenium poisoning. In the case of administered doses of selenium equal to 3200 µg/day, few symptoms of selenium toxicity were observed. Selenium supplementation in patients with rheumatoid arthritis at a dose of 600 µg/day in the form of selenium yeasts showed significant reduction of arthralgia and a lack of side effects [30]. During the winter season in India, the occurrence of chronic selenosis or exacerbation of its symptoms is often observed. Ataxia and dermatological changes are reported [35].

## 5. Physiological Importance of Selenium in Human and Animal Organisms

Selenium is an essential micronutrient, whose trace amounts are essential for life [42,43,44]. It constitutes an integral part of selenoproteins and some antioxidant enzymes such as glutathioneperoxidase (GPx), thioredoxinreductase (TRxR), and iodothyronine deiodinase (DIO), which protect cells from the damaging effects of free radicals produced during oxidation.

Glutathione peroxidase (GSH-Px) plays a protective role against oxidation of lipid membranes. Due to the presence of reduced glutathione (GSH), it catalyzes the reduction of hydrogen peroxide (H_2_O_2_) and organic peroxides (ROOH) forming selenious acid as an intermediate product or the corresponding alcohols (ROH) [45,46].

GSH-Px is highly effective and was also one of the first selenoproteins to be characterized. The majority of the types of GSH-Px are characterized by a tetrameric structure with one selenium atom in each subunit [46,47,48,49]. Five types of GSH-Px have been identified till now and they are classified according to their place of occurrence [50]. Peroxidases commonly occurring in an organism are known as classical or cellular peroxidases (cGSH-Px). Moreover, phospholipid hydroperoxide (PH-GSH-Px) also occurs commonly.

The remaining ones are the most common in locations defined by their name: gastrointestinal (GSH-Px-GI), extracellular or plasma (pGSH-Px) [41,48,51]. Emerging hyperoxide or organic hydroperoxides (ROOH) are reduced by this enzyme in the presence of a reducing agent such as glutathione (GSH). The reaction products are water, the appropriate alcohols, and oxidized glutathione (GSSG). PH-GSH-Px, apart from possessing a different monomeric structure and having broader substrate specificity against reducing agents, exhibits additional functions. The role it plays in the synthesis of prostaglandins should be included herein [52].

Selenium is also a component of other enzymes, particularly iodothyronine deiodinase which catalyzes the deiodonization of thyroxine (T4) to triiodothyronine (T3). Deiodinases play a key role in the regulation of thyroid hormones. They are responsible for the control of proper development, growth, and cell metabolism [53]. In case of selenium deficiency, iodine removal is disrupted, resulting in thyroid gland disorders—the gland responsible, among others, for lipid metabolism and thermogenesis. Therefore, it can be concluded that selenium, like iodine, is an essential element for proper thyroid function [47,54].

Thioredoxin reductase is a selenium-dependent flavoprotein, which reduces oxidized thioredoxin. Thioredoxins are strong electron donors for reducing enzymes, including ribonucleotide reductase and thioredoxin peroxidase. Thioredoxins exhibit activity as growth factors, apoptosis inhibitors and hydroperoxidase reductors. Furthermore, thioredoxin reductase reduces several low-molecular compounds such as oxidized glutathione (GSSG), dehydroascorbic acid, vitamin K, lipid peroxides, and hydrogen peroxide (H_2_O_2_) [55].

Selenium occurs in the composition of active selenoproteins that play an important role in many physiological processes. Selenoprotein P (SEPP1) is involved in defending an organism against the damaging effect of free radicals. It actively participates in the storage and transport of selenium; moreover, it is a good indicator of selenium resources in the organism [56]. SEPP1 exhibits a heavy metals chelator function through the formation of nontoxic selenium–metal complexes [15]. Selenoprotein W prevents excessive oxidation and is involved in muscle metabolism [3]. Selenium is also an essential component of selenophosphate synthetase. This enzyme plays an important role in the synthesis of selenophosphate and catalyzes binding of selenocysteine to selenoproteins.

Selenium additionally plays a key role in the immune system regulation [46]. The element stimulates the immune system to increase the production of antibodies (IgG, IgM) and causes increased activity of T cells and macrophages [42]. The synergistic effect of selenium and vitamin E contributes to a slowdown of the aging process and an increase in the speed of cell regeneration. It is probably the most important nutrient, inhibiting the progression of HIV infection to full-blown AIDS [57]. Moreover, this element exhibits antibacterial and antiviral properties and alleviates the course of disease in patients infected with hepatitis, including hepatitis A (HAV) and hepatitis E (HEV) [58]. Selenium also exhibits protective properties against hepatitis B and C [4]. In addition, the element plays a pivotal role in the transmission of nerve impulses in the central nervous system [4,59]. The anticancer mechanism of selenium is related mainly to its antioxidant activity as commonly known. Despite the essential function of anti-free-radical mechanisms in the antitumor defense mechanisms, the significant impact of this element on the cytotoxic activity of natural killer (NK) cells can be highlighted [56]. Clinical studies have shown that selenium may also protect against the occurrence of prostate, lung, and colorectal cancer [60].

The functions of numerous selenoproteins are still poorly understood due to the scarce literature on the topic. Among these proteins, the following selenoproteins could be distinguished: selenoprotein W (probable function in muscle metabolism), selenoprotein S (control of redox balance in cells), selenoprotein R (probable antioxidant function), and selenoproteins N and M [50,53,60,61]. However, reports on the close relationship between the content of selenium in an organism and certain homeostasis disorders demonstrate the role of selenium also in these aspects [61,62,63,64].

The most effective anticarcinogenic effect is achieved when selenium is administered as a preventive agent prior to onset of a disease or at its early stage of development [61,63]. Diet supplements containing selenium in its composition may decrease the incidence of cancer occurrence [65,66,67]. Selenium occurring in plasma is mainly bound to proteins. For instance, these are GSH-Px, SEPP1, thioredoxin reductase, selenoprotein (Sep15); despite the beneficial role of thioredoxin reductase for the organism, its protective effect towards disease development is doubtful [68].

SEPP1 covers three basic functions: it exhibits antiradical activity, constitutes a storage and transporting selenium protein and exhibits anticarcinogenic properties. Thioredoxin reductase is considered a key enzyme involved in the metabolism of selenium, and is involved in the regulation of the redox state of cells [68].

The greatest importance is attributed to the absorption of selenium in the gastrointestinal tract of organisms. In the intestine, about 85%–95% of selenium quantity supplied with food is absorbed. Bioavailability depends on the form. Organic selenium compounds are absorbed at the level of 90%–95%, while inorganic compounds are less accessible by 10% on average. Immediately after entering into the bloodstream, selenium is bound by red blood cells, albumins, globulins of serum. In this form it is transported to many tissues, it can also penetrate the placenta. Relative large amounts in relation to other tissues are found in the liver, renal cortex, pancreas, thyroid, pituitary, and testis, but are also accumulated in hair and nails. The largest amount of selenium, that is about 50%, is found in the skeletal muscle.

It has been shown that selenium contributes to normal cell growth and has an important role in modulating the action of transcription factors and cell signaling systems. The biological role of selenium is to prevent of diabetes, infertility, cancer, and cardiovascular diseases [69]. It is responsible for the optimum functioning of the endocrine system and is involved in the modulation of inflammatory responses [46]. Moreover, selenium protects against the toxic effects of metals: lead, cadmium, arsenic, mercury, and organic compounds as exemplified by the paraquat herbicide [53].

## 6. Recommended Doses of Selenium

Review of the literature data [70,71] reveals that the recommended intake for selenium varies depending on the geographical region. Residents of the Czech Republic consume the least selenium (dose estimated at 10–25 µg/day), while residents of Venezuela (200–350 μg/day) and selected areas of China (7–4990 µg/day) consume the highest doses. According to the European Food Safety Authority [72], daily intake of selenium in the European population is estimated between 20 and 70 μg [73,74,75,76,77]. The level of selenium intake in Poland ranges from 30 to 40 µg/day [70]. In Spain, the intake of selenium is 44–50 µg/day, in Austria it is 48 μg/day, while in Great Britain it is 34 µg/day [30,78]. The WHO recommends a daily dose of selenium in an amount between 30 and 40 µg for adult individuals, emphasizing that doses of selenium up to 400 µg/day are safe. According to the Food and Nutrition Board of the National Academy of Science, the daily requirement for selenium depending on the age varies in men and women between 40–70 μg and 45–55 μg, respectively [22,78]. During pregnancy and lactation, selenium intake should be established at 60–70 μg/day [77].

Studies on the selenium contents in plants show that deficiency of selenium among animals is quite common, especially in areas where deficiency of this trace element is observed [6,7,8]. Therefore, selenium has to be supplemented in the diet of animals, which has become an important issue. Recommended intake doses are different depending on the species. On average, a recommended supplementation at levels of 0.1–0.3 mg/kg dry weight is accepted [79]. Among the supplementation methods for animals, the following should be distinguished: enrichment of fodder crops, addition of selenates directly to fodder, and direct injection of selenium to animals [76]. Studies on the use of yeast enriched with selenium as components of feed are also interesting [1,34,48,80,81,82]. In cattle feeding, an appropriate concentration of selenium for daily intake should be specified. One defined indicators, which properly cover the demand for this element. An indicator of selenium supply is its concentration in the blood serum, which in adult cattle should range between 0.07 and 0.10 μg/mL. A total of 0.025 μg/mL was established as the limit of acceptable deficiency in cattle [79].

## 7. Methods of Dietary Supplementation with Selenium

The primary source of selenium is a properly selected and balanced diet, in which food products cover the demand for this element. To meet societal demands, one can enrich fermented products with micronutrients essential for the proper development of the organism [80]. Among individuals at high risk of selenium deficiency, patients with phenylketonuria are mentioned [83]. The required modifications of the diet in terms of amino acid composition may result in the lack of selenoamino acids – an important source of selenium. Exposure to selenium deficiency was also reported in other diet-related diseases and malabsorption, as well as in patients fed parenterally and in metabolic diseases. Patients who have undergone radio- and chemotherapy are another group with deficiency of this micronutrient [1,34,84].

Food supplementation with selenium should be carried out in a careful and controlled way, to avoid causing the opposite effect than intended, because selenium is one of the most toxic elements in relatively small quantities being at the same time an essential micronutrient with an important biological role. The range between the necessary quantity of selenium and toxic dose is very narrow [1,6].

Enrichment of food with compounds raising its nutritional value *i.e.*, with selenium, may be conducted using classical methods. The classical method of diet supplementation with selenium can be carried out indirectly—through fertilization of soils with selenium compounds in order to obtain plants enriched with this element or through enrichment of fodder with selenium compounds to obtain, e.g., eggs with more selenium. In contrast, a direct method of dietary supplementation with selenium is based on the intake of dietary supplements which constitutes a source of this element [1,48,85].

Selenium deficiencies are a critical problem worldwide, with negative impacts on health and lifespan. Biofortification is the process by which the nutritional quality of food is improved through an agricultural approach that can improve human nutrition on the world. Biofortification of agricultural crops with Se, by means of adding Se along with fertilizers, is a useful technique to increase the consumption of Se by animals and man [76].

Lately, great interest in alternative forms of supplementation has been observed. Therefore, the direction towards the use of microorganisms for the production of functional foods seems to be justified. An interesting supplementation strategy is to use the functional foods. These may be products derived from plant biomass enriched with selenium, fermented food containing lactic acid bacteria as well as yeast accumulating significant quantities of selenium [86].

Based on this information and the fact that the basis of the food pyramid constitutes grain products such as baker’s goods, bread seems to be an appropriate product to apply supplementation. Produced using sourdough with addition of bacteria and yeasts enriched with selenium, it is able to safely increase the level of selenium in the diet without any decline in the quality in relation to standard bread [87].

The source and chemical form of this element are gaining increasing importance in theoretical considerations on dietary supplementation with selenium. Selenium yeasts are effective and safe sources of selenium (selenomethionine) and represent a better absorbed form of this element, whose absorption is enhanced by vitamins present in the yeast biomass (mainly vitamins B and E) [88]. Animals unlike plants and microorganisms cannot synthesize selenomethionine themselves. However, they are capable of synthesizing selenocysteine from both inorganic and organic selenium compounds. The latter transformation occurs as a result of the transfer of selenium atom from selenomethionine to serine, similarly as in case of sulfur amino acids, methionine and, cysteine [43]. To a great extent, selenium metabolism, due to its chemical similarity to sulfur, occurs via the same or similar pathway [43]. Selenoamino acids, mainly SeMet, can nonspecifically be incorporated into proteins replacing sulfur amino acids [20,43,44]. Then, they constitute a pool, unlike all remaining forms of selenium, which is subjected to continuous exchange. Therefore, one of the most easy-to-absorb selenium form is selenomethionine [1].

Selenium occurring in the form of selenomethionine is characterized by the highest bioavailability as compared to the inorganic forms [1,11]. It is conditioned by the nonspecific incorporation of this amino acid in certain protein molecules of higher organisms. The accumulation and retention of selenium originating from selenium yeast by the human organism is estimated between 75% and 90% [11].

In many countries, innovative technological process based on the production of selenium-enriched food products such as eggs, meat, milk, has been successfully introduced [6,89]. Pork or poultry enriched with selenium is available in Korea, while eggs enriched with this element are present on the market in 25 countries around the world. When analyzing scientific reports in terms of the production of functional foods, it seems obvious that selenium-enriched eggs may be used in supplementation of micronutrient deficiencies and maintenance of metabolic balance of the organism [90].

One egg or poultry meat supplemented with selenium in an amount of 100 g can provide 50% of the daily requirement for this element [90,91]. Currently, many research centers in the world have been working on obtaining new food products which constitute sources of selenium. The most desirable feature is to provide organic forms of selenium with the highest bioavailability in the ready-to-use product. The use of supplements containing organic selenium of yeast origin in terms of deficiency exhibits multiple beneficial effects on human health [81,92]. However, one should keep in mind that the fundamental source of selenium for the human organism is always an appropriate and balanced diet. Yeast preparations containing selenium could favor the supplementation of the daily diet in this element. This will enhance the consumer attractiveness of food product, especially for those individuals who are interested in purchasing foods supplemented with micronutrients [1].

## 8. Influence of Selenium in Cancer Prevention

Current knowledge on the diversity of selenium compounds present in food of animal and vegetable origin indicates that selected compounds exhibit a strong anticancer effect, for example, Se-methylselenocysteine and γ-glutamyl derivatives [58]. Identification of the diversity of selenium compounds present in the food products of animal and vegetable origin and determination of their impact on human health are, therefore, problems that require continuous research [42]. In 1998, selenium was considered an active compound in “functional foods” [93], and the study results collected during recent years have confirmed its beneficial role in protection against the formation and development of different diseases and neoplasms [94,95].

Se-methylselenocysteine is one of the active organic compounds occurring in selenium yeast cells. The anticancer effect of Se-methylselenocysteine depends on the expression of β-lyase. Little information is available on the expression of these very interesting classes of enzymes in cancer. SeMet supplementation can significantly reduce the incidence of metastasis and tumors in lungs and influences the reduction of tumor size in mice. Supplementation with a mixture of soy proteins characterized by high selenium content has also demonstrated similar results [96].

Knowledge about mostly selenium compounds is still insufficient. Selol is a new organoselenium compound synthesized at the Medical University of Warsaw. This is just one of several examples of very recent and promising developments and closely related research fields (medicine, biochemistry, biology). Selol is a semi-synthetic compound containing selenite. It is a mixture of selenitetriglycerides obtained by incorporating selenic acid (IV) into molecules of fatty acids from plant oil. Selol attacks all vitally important mechanisms of metabolism of cancer cells. Using it in treatment may decrease the risk of lifestyle diseases such as cancer, cardiovascular diseases, multiple sclerosis, diabetes, rheumatism and many other diseases caused by oxidative stress and redox state disorder in cells [97]. Studies presented by Flis *et al.* [98] showed that Selol is non-toxic and non-mutagenic. It exhibits strong anti-cancer activity *in vitro* in many cancer cell lines. The potential to use Selol as a prophylactic and therapeutic source of selenium requires further work [99]. The preparation is currently thoroughly studied in several research centers in Poland [97,100].

Numerous studies have demonstrated the benefits of selenium supplementation in the treatment of neoplasms and autoimmune thyroid disorders. Selenium supplementation appears to potentiate the activity of selenoproteins, reducing the local inflammatory reactions that inhibit the formation of anti-TPO, and has a beneficial impact on thyroid gland morphology. In Graves’ disease, the administration of selenium can contribute to the promotion of euthyroid [42].

The protective effects of selenium in the etiology of cancer diseases result from its effect on cell membranes protecting against oxidative stress, and also from the stabilizing effect on DNA and enhancement of cellular immune response [1,87]. It has also been found that selenium inhibits tumor cell proliferation via the effect exerted on the expression of p53 tumor suppressor gene, and Bcl-2 apoptosis-suppressor gene [101]. It has been shown that in the case of cancer occurrence, lesions in the *p53* gene are reported [102]. It should be emphasized that the anticarcinogenic effect of selenium depends on the chemical compound of the element administered, its dosage, and type of agent that induces the development of cancer [102,103,104]. In addition, *p53* gene plays an important role in the processes of control and regulation of cell lifecycle—DNA replication—whose activity depends on the concentration of selenium in plasma [105].

Among the inorganic compounds which are used, one can mention selenite (IV), and in the case of organic compounds, selenomethionine, Se-methylselenocysteine, and others can be distinguished. Although administered selenium compounds exhibit different protective effects they are metabolized to a final product (methylselenol) which exerts the most potent anticarcinogenic effect [106,107].

It has been found that in individuals suffering from lung, prostate, liver, and stomach cancer, the level of selenium content in plasma was decreased in comparison to healthy individuals [70]. Nipple cancer in women in whom there was no correlation between the selenium status in the organism and disease development, constituted an exception [49]. It is probably related to the specific structure exhibiting little correlation with other parts of the organism [71]. Expression of some selenoproteins, especially SEPP1, requires the supplementation of larger amount of dietary selenium to an organism, e.g., in the form of selenium yeasts [56,108,109].

The concentration of selenium in the thyroid gland is higher in comparison to other tissues. The presence of low levels of selenium in serum of patients reflected the probability of increased risk of thyroid cancer development. Selenium exhibits anticancer properties, furthermore it prevents the formation of neoplastic lesions in various organs and tissues, as shown in numerous literature reports [105,110,111]. Many studies have shown that there is an association between geographical distribution of selenium in the soil, its amount consumed in the diet, and the incidence and mortality of individuals due to cancer developed in various organs [68].

Furthermore, a significant number of individuals may have higher selenium requirements during the process of selenoprotein synthesis. These individual differences in the levels of expression of selenoproteins may be associated with a single nucleotide polymorphism found in genes encoding selenoproteins and determining the effectiveness of selenium incorporation into the structures of selenoproteins [61,112]. When selenium binds to the cellular structures of proteins, it causes conformational changes [86]. Furthermore, the modified proteins affect the decrease of enzymes’ activity involved in metabolic processes of cancer cells [113].

In the study conducted by Jönsson-Videsäter *et al.* [68], the effect of sodium selenite (IV) on the induction of apoptosis in lung cancer cell lines—U-1285 which is sensitive to doxorubicin and U-1285-dox that is nonsensitive to the cytotoxic drug activity—was evaluated. An iincreased number of apoptotic cells in the U-1285-dox cell lines in comparison to U-1285 cell line was observed, which was related to increased activity of thioredoxin reductase – selenoproteins involved in redox reactions. In the described cell lines, no effect of sodium selenite (IV) on the activity of caspase 3 was observed, which suggests that the increased number of apoptotic cells was independent of caspase activation [42,114]. However, the role of selenoproteins and mechanisms involved in these processes are still not clear.

Selenium levels in plasma, and particularly the concentration of selenoprotein P, are indicators of selenium status in the organism. In males, low levels of selenium are accompanied by higher probability of occurrence of lung and prostate cancers [65,104]. In terms of elderly people, selenium plays an important role in maintaining health status and immune response [95]. However, the role of selenoproteins and mechanisms involved in these processes are still not clear.

According to the Nutritional Prevention of Cancer indications, a dose of 200 µg Se/day in the form of selenium yeasts decreases the risk of colon, rectal, prostate, and lung cancers [107,109]. Moreover, supplementation of selenium at the same dose in the diet significantly reduced the risk of stomach cancer in individuals with low selenium status [7]. It was found that selenium-enriched broccoli maybe more effective in inhibiting the formation of colon tumors. Furthermore, they improve the activity of GPx in epithelial cells [114]. It has been demonstrated that selenium-enriched genera, such as *Allium* and *Brassica*, reduce the risk of colon cancer [107].

Other studies have shown that SeO_2_ affected the expression of *p53* and *Bcl-2* genes in three lung cancer cell lines: A549, GLC-82, and PG. It has been observed that with increasing concentration of SeO_2_, concentration of Bcl-2 protein decreased, but only in the case of A549 cell line, while the level of p53 protein increased in all three cell lines [94].

Selenium exhibits the ability to activate genes whose expression results in the formation of the enzymes involved in phase II xenobiotic metabolizing pathway – the detoxification stage, and slows down the synthesis of enzymes of phase I [42]. Regardless of the cell type, selenium inhibits cell division in the G1 phase of the cell cycle, inhibiting the gene expression of cyclin A, cyclin D1, CPC25A protein, CDK4 protein, PCNA [proliferating cell nuclear antigen] protein, E2F protein, while increasing the expression of genes of P19, P21 proteins, superoxide dismutase, glutathione S-transferase. It has also been found that selenium inhibits the synthesis of osteopontin—a significant protein in the formation of metastases [46].

Many reports are available in the literature that demonstrate the effect of selenium in carcinogenesis and cancer treatment. However, very fewer studies have focused on the subsequent processes of tumor progression and the occurrence of new tumor foci. Taking into account that metastasis is a common cause of death in cancer patients, large-scale clinical trials that focus on the effect of selenium in cancer prevention should be conducted.

The results of the studies presented by Kenfield *et al.* [110] have showed that selenium supplementation at a dose of 140 µg/day or more may increase the risk of death after diagnosis of metastatic prostate cancer. Therefore, particular caution should be paid in terms of the use of such supplements among men with prostate cancer. In contrast, the studies presented by Heras *et al.* [115] have shown that selenium plays a significant role in preventing high-grade tumors. Sep15 and TrxR1 (thioredoxin reductase 1) proteins are of particular importance.

The discovery of essentiality of selenium compounds for animal and human organisms has contributed to the development of medical knowledge on this element [59]. Many functions of selenium have been recognized. Most of them are fulfilled by selenium-dependent enzymes. It is amazing how the addition of one atom to the enzyme structure can interact with complex biochemical pathways, thus determining the variety of functions and at the same time prejudging its indispensability for the organism.

## 9. Conclusions

Selenium is an essential element for the proper functioning of human and animal organisms. Unlike other trace elements, selenium is an element with a very narrow quantitative range of concentrations between deficiency, physiological status, and toxic dose. A lot of data indicate insufficient coverage in terms of the demand for selenium in humans and animals due to its low content of food products (daily diet). This confirms that compounds of this element should be taken as a daily supplement. Evaluating the effectiveness of this element as a daily supplement constitutes a subject of comprehensive research, which explains the influence of the chemical form of this compound on the metabolism and health of all organisms, when introduced as a daily supplement. Searching for food constituents enriched with selenium, exhibiting anticancer properties, and explaining their mechanisms of action in metabolism can reduce the incidence of cancer and autoimmune diseases. Further research should focus on the anticancer effect of selenium and dietary supplements rich in this element.

## Figures and Tables

**Table 1:** Selenium content in selected food products (fresh weight).

Food Products	Selenium Content (µg/g)	Reference
Selenium yeast	3000	[12]
Brazil nuts	0.85–6.86	[7]
Garlic	0.5	[13]
Onion	0.5	[7]
Salmon	0.21–0.27	[14]
Eggs	0.17	[15]
Beef	0.35–0.47	[16]
Chicken	0.57	[17]
Milk products	0.01–0.55	[7]

**Table 2:** The structures, names, molecular formula and abbreviations of Se compounds referred to in this review.

Name	Molecular Formula	Chemical Structures	Abbreviation
Sodium Selenite (IV)	Na_2_SeO_3_	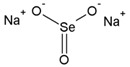	Se(IV)
Sodium Selenate (VI)	Na_2_SeO_4_	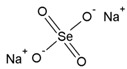	Se(VI)
Selenomethionine	C_5_H_11_NO_2_Se	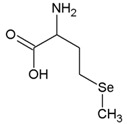	SeMet
Selenocysteine	C_3_H_7_NO_2_Se	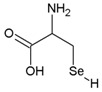	SeCys
γ-glutamyl- Se-methylselenocysteine	C_9_H_16_N_2_O_5_Se	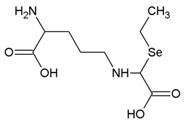	γ-glutamyl- SeMeSeCys
Se-methyl selenocysteine	C_4_H_9_NO_2_Se	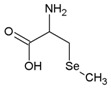	SeMeSeCys
Methylselenol	CH_4_Se	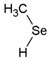	MeSeH
